# The draft genome of *Actinia tenebrosa* reveals insights into toxin evolution

**DOI:** 10.1002/ece3.5633

**Published:** 2019-09-18

**Authors:** Joachim M. Surm, Zachary K. Stewart, Alexie Papanicolaou, Ana Pavasovic, Peter J. Prentis

**Affiliations:** ^1^ Faculty of Health School of Biomedical Sciences Queensland University of Technology Kelvin Grove Qld Australia; ^2^ Institute of Health and Biomedical Innovation Queensland University of Technology Kelvin Grove Qld Australia; ^3^ Science and Engineering Faculty School of Earth, Environmental and Biological Sciences Queensland University of Technology Brisbane Qld Australia; ^4^ Institute for Future Environments Queensland University of Technology Brisbane Qld Australia; ^5^ Hawkesbury Institute for the Environment Sydney NSW Australia

**Keywords:** Cnidaria, concerted evolution, sea anemone, venom

## Abstract

Sea anemones have a wide array of toxic compounds (peptide toxins found in their venom) which have potential uses as therapeutics. To date, the majority of studies characterizing toxins in sea anemones have been restricted to species from the superfamily, Actinioidea. No highly complete draft genomes are currently available for this superfamily, however, highlighting our limited understanding of the genes encoding toxins in this important group. Here we have sequenced, assembled, and annotated a draft genome for *Actinia tenebrosa*. The genome is estimated to be approximately 255 megabases, with 31,556 protein‐coding genes. Quality metrics revealed that this draft genome matches the quality and completeness of other model cnidarian genomes, including *Nematostella*, *Hydra*, and *Acropora*. Phylogenomic analyses revealed strong conservation of the Cnidaria and Hexacorallia core‐gene set. However, we found that lineage‐specific gene families have undergone significant expansion events compared with shared gene families. Enrichment analysis performed for both gene ontologies, and protein domains revealed that genes encoding toxins contribute to a significant proportion of the lineage‐specific genes and gene families. The results make clear that the draft genome of *A. tenebrosa* will provide insight into the evolution of toxins and lineage‐specific genes, and provide an important resource for the discovery of novel biological compounds.

## Graphical abstract

To date, the majority of studies characterizing toxins in sea anemones have been restricted to species from the Actinioidea superfamily; however, no draft genomes are currently available for this superfamily. Here we have sequenced, assembled, and annotated the first Actinioidean draft genome for *Actinia tenebrosa*. The results make clear that the draft genome of *A. tenebrosa* will provide insight into the evolution of toxins, species‐specific genes, the cnidarian, and hexacorallian core‐gene set, and provide an important resource for novel biological compounds.

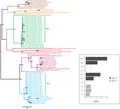

## 1 INTRODUCTION

Cnidarian venom consists of a diverse array of peptides that have distinct biochemical and pharmacological properties (Frazão, Vasconcelos, & Antunes, 2012; Jouiaei, Sunagar, et al., 2015). These toxins are used for a variety of different roles, consistent with nematocyst morphology and function (Beckmann & Özbek, 2012; Fautin, 2009; Fautin & Mariscal, 1991; Kass‐Simon & Scappaticci, 2002; Özbek, 2010). Multiple toxin types have been pharmacologically characterized in cnidarians, including neurotoxins, pore‐forming toxins, and enzymatic toxins (Casewell, Wüster, Vonk, Harrison, & Fry, 2013; Daly, 2016; Jouiaei, Sunagar, et al., 2015; Jouiaei, Yanagihara, et al., 2015; Prentis, Pavasovic, & Norton, 2018). Consistent with other venomous lineages, cnidarian venoms are a rich source of novel biological compounds, often being encoded by genes that lack homology to sequences other than cnidarians (Moran, Praher, et al., 2012; Sebé‐Pedrós et al., 2018; Sunagar et al., 2018; Surm et al., 2019).

Recent studies have revealed a high frequency of cnidarian‐specific genes is enriched within the cnidocyte (Sebé‐Pedrós et al., 2018; Sunagar et al., 2018). Many of these cnidarian‐specific genes expressed in the cnidocytes encode for toxin peptides (Columbus‐Shenkar et al., 2018; Sebé‐Pedrós et al., 2018). This highlights that cnidarians possess both morphological and biochemical novelties and that the evolution of these innovations may be related. This is consistent with evidence that acrorhagin 1 and acrorhagin 2 are novel toxin‐coding genes which are localized to the acrorhagi, a morphological structure used for envenomation that is unique to sea anemones from Actinioidea (Honma et al., 2005; Macrander, Brugler, & Daly, 2015).

Indeed, understanding the evolution of venom and its delivery in cnidarians can provide insights into the innovation of morphological and biochemical novelties. While the majority of cnidarian toxin research has focussed on sea anemones from the Actinioidea superfamily (Prentis et al., 2018), no highly complete sequenced genomes for members of this superfamily currently exist (Urbarova et al., 2018). This lack of genomic resources limits our collective ability to understand the phylogenetic and molecular evolutionary histories of toxin‐encoding genes within this superfamily. Such a resource would provide an excellent model to investigate the evolution of novel morphological and cellular structures, and their relationship with novel genes.

*Actinia tenebrosa* is a sea anemone from the superfamily Actinioidea. This species is similar in morphology to the northern hemisphere species, *Actinia equina* (Farquhar, 1898; Sherman, Peucker, & Ayre, 2007; Watts, Allcock, Lynch, & Thorpe, 2000), both of which have been used as model organisms for the investigation of sea anemone toxins (Honma et al., 2005; Maček & Lebez, 1988; Minagawa, Sugiyama, Ishida, Nagashima, & Shiomi, 2008; Moran et al., 2008; Norton, Maček, Reid, & Simpson, 1992; O'Hara, Caldwell, & Bythell, 2018; Prentis et al., 2018; Surm et al., 2019; Watts et al., 2000). Here, we have assembled and annotated the first draft genome for *A. tenebrosa*. We performed phylogenomic analyses and provide insights into the evolution of lineage‐specific genes in cnidarians, specifically revealing that these novel genes undergo increased rates of expansions compared with gene families that have a wider taxonomic distribution. Moreover, genetic innovations restricted to Actinioidea are found to be enriched for functions related to venom and its delivery. The suite of toxin and toxin‐like (TTL) genes identified in *A. tenebrosa* reveal an abundance of gene families evolving through lineage‐specific duplications and, in some cases, concerted evolution. This study shows that gene duplication and divergent selective pressures have shaped the genetic variation in genes encoding toxins in actiniarians.

## 2 METHODS

### 2.1 Genome assembly of *Actinia tenebrosa*

#### 2.1.1 Sample preparation, sequencing, and assembly

Samples of *A. tenebrosa* were collected from the intertidal zone at Coolum, (QLD, Australia). Tissue from a single individual was used to extract high‐quality gDNA using the E.Z.N.A. Mollusc DNA Kit (Omega Bio‐Tek; Stefanik, Wolenski, Friedman, Gilmore, & Finnerty, 2013). Extracted gDNA was used to construct four paired‐end (PE) libraries sequenced on Illumina 2500 HiSeq platform using multiple insert sizes (170, 500, 2,000, 5,000 bp) with a read length of 100 bp (NCBI BioProject PRJNA505921). Sequencing resulted in over 150 million PE reads per library, with over 96% being high‐quality (*Q* > 30, [*N*(ambiguous bases) < 1%]). Contiguous sequences were generated and scaffolded using a manual operation of ALLPATH‐LG (Butler et al., 2008) with a focus on removing redundant sequences.

The presence of the complete mitochondrial genome of *A. tenebrosa* in the draft genome was investigated. Assembled contigs were queried using BLASTN against a database which consisted of the complete mitochondrial genome of *A. equina*. Contigs receiving a significant hit (*e* value 1*e* ^−05^) were imported into Geneious 9.1.6 and aligned using a global alignment with free end gap and 100% identity. This resolved a single sequence, of 20,691 bp, and was aligned to the complete mitochondrial genome of *A. equina* using eight iterations of MUSCLE. Gene order and annotation of the mitochondrial genome of *A. tenebrosa* were performed as per Wilding and Weedall (2019).

#### 2.1.2 Annotation

##### Repeat library generation

Homology and ab initio‐based methods were used to identify repeat regions and low‐complexity DNA sequences. Miniature Inverted‐repeat Terminal Elements (MITEs) were predicted with MITE‐HUNTER v.11‐2011 (Han & Wessler, 2010) and detectMITE v.20170425 (Ye, Ji, & Liang, 2016). MITE predictions were clustered using CD‐HIT v.4.6.4 (Fu, Niu, Zhu, Wu, & Li, 2012). Parameters = “cd‐hit‐est ‐c 0.8 ‐s 0.8 ‐aL 0.99 ‐n 5” (same parameters used by detectMITE). Prediction of long terminal repeat retrotransposons (LTR‐RTs) was performed using LTRharvest (GT 1.5.10; Ellinghaus, Kurtz, & Willhoeft, 2008) and LTR_FINDER v.1.06 (Xu & Wang, 2007), and these results were combined using LTR_retriever commit 9b1d08d (Ou & Jiang, 2018) to identify canonical and noncanonical (i.e., non‐TGCA motif) LTR‐RTs. MITE and LTR‐RT libraries were concatenated, and the genome sequence was masked using RepeatMasker open‐4.0.7 (Smit, Hubley, & Green, 2013) with settings “‐e ncbi ‐nolow ‐no_is –norna.” De novo repeat prediction was performed using RepeatModeler open‐1.0.10 (Smit & Hubley, 2008) with the masked genome as input.

All repeat models were curated to remove models putatively part of protein‐coding genes. Any models confidently annotated by LTR_retriever or RepeatModeler (i.e., not classified as “Unknown”) were removed from consideration as they are not likely to be part of protein‐coding genes. Open reading frames from the remaining repeat models were extracted and examined using HMMER 3.1b2 (Eddy, 2011) to identify models that only contained domains associated with transposable elements. For this purpose, we collated a list of transposon‐associated domains which primarily consisted of domains identified by Piriyapongsa, Rutledge, Patel, Borodovsky, and Jordan (2007) with additional Pfam (Finn et al., 2014) and NCBI CDD (Marchler‐Bauer et al., 2015) domains included on the basis of manual inspection of domain prediction results for putative transposable elements. Repeat models that contained a TE‐associated domain prediction were removed from consideration and assumed to be true‐positives. A custom database of known genes was created to enable BLAST comparison of remaining repeat models and subsequent removal of false predictions from protein‐coding genes. The database includes the UniProtKB/Swiss‐Prot proteins as well as the gene models of *Nematostella vectensis* (v.2.0; Putnam et al., 2007; Schwaiger et al., 2014), *Exaiptasia pallida* (v.1.1; Baumgarten et al., 2015), *Acropora digitifera* (v.0.9; Shinzato et al., 2011), and *Hydra vulgaris* (Chapman et al., 2010). This database had probable transposons removed via the same process detailed above using HMMER 3.1b2 and domain organization. Any remaining repeat models were removed from the initial custom repeat library (CRL) if they had significant BLASTX hits (*e* value < 1*e* ^−02^) when queried against the gene model database. The final curated CRL was used to soft‐mask the *A. tenebrosa* genome using RepeatMasker (‐e ncbi ‐s ‐nolow ‐no_is ‐norna ‐xsmall) for later gene prediction. Scripts were produced to automate this process and are available from https://github.com/zkstewart/Genome_analysis_scripts/tree/master/repeat_pipeline_scripts.

### 2.2 Gene model prediction and annotation

Following the masking of repeat regions, gene models were predicted using ab initio methods guided by transcriptional expression. These reads included the Red and Brown ecotypes obtained from NCBI (Bioproject PRJNA313244; van der Burg, Prentis, Surm, & Pavasovic, 2016). Raw reads were quality trimmed using Trimmomatic (Bolger, Lohse, & Usadel, 2014) with parameters used by the Trinity de novo assembler (Haas et al., 2013; MacManes, 2014). Trimmed sequences were aligned against the genome using STAR 2.5 (Dobin et al., 2013) using the two‐pass procedure for the de novo identification of transcription splice sites. The SAM file produced by STAR was converted to BAM format and sorted using samtools v.1.5 (Li et al., 2009). Gene models were predicted by BRAKER1 v1.11 (Hoff, Lange, Lomsadze, Borodovsky, & Stanke, 2016) using the soft‐masked genome assembly and the STAR alignment file as inputs. The completeness of the protein‐coding genes was then assessed using BUSCO (Simão, Waterhouse, Ioannidis, Kriventseva, & Zdobnov, 2015; Waterhouse et al., 2018).

Gene models were annotated by querying models against the Uniclust90 database (Mirdita et al., 2017) using MMseqs2 with an *e* value < 1*e* ^−05^ (Steinegger & Söding, 2017). Gene Ontology (GO) terms associated with the representative UniProtKB sequence for each Uniclust90 hit were attributed to the *A. tenebrosa* gene model using the idmapping_selected.tab file provided by UniProtKB. Protein domain predictions were performed by HMMER 3.1b2 using a custom domain database, which included NCBI's CDD in addition to CATH (S35 v.4.1.0; Dawson et al., 2017) and SUPERFAMILY (1.75; Gough, Karplus, Hughey, & Chothia, 2001), and tabulated using scripts available from https://github.com/zkstewart/Genome_analysis_scripts/tree/master/annotation_table.

### 2.3 Gene family evolution

Using translated gene models from *Nematostella vectensis*, *Exaiptasia pallida*, *Acropora digitifera*, *Amplexidiscus fenestrafer*, *Discosoma* sp., and *Hydra vulgaris*, an “all‐against‐all” BLASTP analysis (*e* value < 10*e* ^−5^) was performed. ORTHOMCL version 2.0.9 (Li, Stoeckert, & Roos, 2003) was used, with default parameters, to assign proteins into orthologous gene groups. Phylogenetic analyses were performed using single‐copy orthologs (SCO) for each species. A total of 1,314 SCO were identified and aligned using clustal‐omega (Sievers et al., 2011). The alignments were the concatenated, and the best evolutionary mode protein model (JTT+F+I+G4) was determined. Finally, a maximum‐likelihood tree with 1,000 ultrafast bootstrap replicates was generated using IQ‐TREE (Nguyen, Schmidt, von Haeseler, & Minh, 2015).

Following the generation of a cnidarian species tree, the gain and loss of gene families across Cnidaria were inferred using the DOLLOP program from the PHYLIP package version 3.696 (Felsenstein, 1989; http://evolution.genetics.washington.edu/phylip.html). The species tree and a presence/absence matrix of gene families were imported into the DOLLOP program. The most parsimonious evolutionary scenario for the gain and loss of gene families was estimated using Dollo's parsimony law, which assumes genes arise once on the evolutionary tree and can be lost independently in different evolutionary lineages (Farris, 1977). The predicted proteomes from cnidarian species with sequenced genomes were used to investigate the evolution of protein domains. Protein domains were predicted using HMMER 3.1b2 against the Pfam database (*e* value < 1*e* ^−05^), the best hit was retained, and overlapping domains were removed. A Fisher exact test was performed to determine Pfam enrichment with *p*‐value of .05.Finally, we investigated the proportion of shared and unique gene families in actiniarian species. A BLASTP analysis (*e* value < 1*e* ^−05^) was performed with OrthoVenn (Wang, Coleman‐Derr, Chen, & Gu, 2015) using gene models from *A. tenebrosa*, *N. vectensis*, and *E. pallida* to determine the number of shared and unique gene families in each species.

The presence of toxin and toxin‐like (TTL) genes was investigated in *A. tenebrosa*. The TTL genes were identified as per Surm et al. (2019). Briefly, BLASTP was performed against the against the manually curated Swiss‐Prot database (*e* value < 1*e* ^−05^). Significant queries with top BLAST annotations from sequences in the Tox‐Prot database (Jungo & Bairoch, 2005) were considered candidate proteins. Candidate proteins were further interrogated and required to contain a signal peptide identified using SignalP (Petersen, Brunak, Heijne, & Nielsen, 2011).

The phylogenetic and evolutionary histories of multiple toxin gene families were investigated. Candidate sea anemone sodium channel inhibitory toxin (NaTx), sea anemone type 1 potassium channel toxin (KTx), sea anemone type 3 (BDS‐LIKE) KTx, and membrane attack complex/perforin (MACPF) sequences were used for phylogenetic analysis to determine their distribution among cnidarian taxa and aligned to functionally characterized sequences (Jouiaei, Sunagar, et al., 2015; Sunagar & Moran, 2015). The florescent protein (FP) gene family was also investigated to explore the evolution of nontoxin gene families. Sequences were identified in cnidarian genomes by the presence of GFP PFAM domain (PF01353) and aligned to sequences used in previous studies (Alieva et al., 2008; Ikmi & Gibson, 2010).

Protein alignments of candidate gene families were imported into IQ‐TREE (v1.4.2; Nguyen et al., 2015) to determine a best fit of protein model evolution. Phylogenetic trees were generated from the alignments using 1,000 ultrafast bootstrap iterations and visualized using Figtree (v1.4.3, http://tree.bio.ed.ac.uk/software/figtree/). Selection analyses were performed on these gene families using previously published methods (Jouiaei, Sunagar, et al., 2015; Sunagar & Moran, 2015).

## 3 RESULTS

### 3.1 Genome assembly

Using a whole‐genome shotgun strategy, we sequenced and assembled the genome of *A. tenebrosa*. A total of 1.2 billion paired‐end reads, with a length of 100 bp, were sequenced across four different insert size libraries (170, 500, 2,000, and 5,000 bp; Table S1). Raw reads were used to assemble the *A. tenebrosa* genome using ALLPATHS‐LG. The genome size of *A. tenebrosa* is estimated to be ~255 Mbp (Table 1). The draft genome assembled is of similar quality to other cnidarian genomes (Table 1). Although the assembly resulted in the scaffold and contig N50 lower than other cnidarian genomes, the predicted genome completeness using metazoan Augustus gene models is among the highest (89.6%) for cnidarian genomes, with only *N. vectensis* having a more complete assembly (91.6%). The assembly contains ~19% repetitive DNA sequences, which is similar to reported values for other cnidarians (Tables 1 and S2).

**Table 1:** Comparative genome metrics across Cnidaria

Annotation metrics	ADIG	AFEN	ATEN	DSPP	EPAL	NVEC	HVUG
Genome size (Mbp)	420	350	255	428	260	329/450	1,300
Assembly size (Mbp)	419	370	238	444	258	356	852
Total contig size (Mbp)	365	305	206	364	213	297	785
Total contig size (% of assembly)	87	82.43	86.56	81.98	82.5	83.4	92.2
Contig N50 (kbp)	10.9	20	8.4	18.7	14.9	19.8	9.7
Scaffold N50 (kbp)	191	510	159	769	440	472	92.5
Percent repetitive DNA	13	30.7	19.57	37.8	26	26	57
BUSCO (%)	74.7	83.7	89.6	86.3	87.3	91.6	77

Abbreviations: ADIG, *Acropora digitifera*; AFEN, *Amplexidiscus fenestrafer*; ATEN, *Actinia tenebrosa*; DSPP, *Discosoma* sp.; EPAL, *Exaiptasia pallida*; HVUG, *Hydra vulgaris*; NVEC, *Nematostella vectensis*.

### 3.2 Functional annotation of predicted gene models

The ab initio gene model prediction identified 31,556 protein‐coding genes in *A. tenebrosa*. All gene models were validated, receiving significant BLAST hits against multiple *A. tenebrosa* transcriptomes (van der Burg et al., 2016). Our ab initio gene model prediction was highly complete compared with other cnidarian genomes, increasing the previous BUSCO score to 94.6% (Table 1). Only *E. pallida* gene models were more complete (94.7%). Of the 31,556 protein‐coding genes, 19,022 and 25,478 returned a significant BLAST hit (*e* value 1*e* ^−05^) against the Swiss‐prot and TREMBL database, respectively. This highlights that ~80% of the predicted proteome shares sequence similarity to known protein sequences, with ~20% having no similarity to other proteins. In contrast, only 6.56% of *E. pallida* predicted proteome returned no hits to known proteins at this stringency. However, other cnidarian genomes returned similar levels of novelty, with *Discosoma* sp. having 16.17% of proteins returning no hits. The annotation of protein domains revealed 19,056 (~60%) gene models contained identifiable Pfam domains. This is less than other sea anemone genomes, with 78.64% and 68.35% of *E. pallida* and *N. vectensis* gene models having a protein domain, respectively. Additionally, both corallimorpharians genomes reported less than 60% of gene models to encode proteins with known protein domains. Taken together, these results highlight that the draft genome of *A. tenebrosa* is mostly complete, yet a significant proportion of its genes are unique (Table 2).

**Table 2:** Functional annotation of gene models from seven cnidarian species

Annotation metrics	ADIG	AFEN	ATEN	DSPP	EPAL	HVUG	NVEC
BUSCO (%)	80.5	72.8	94.6	68.6	94.7	91.5	93.8
Protein‐coding genes	33,878	21,372	31,556	23,199	26,087	21,990	24,780
SP annotation	24,094	12,959	19,022	13,562	20,515	15,923	18,974
SP annotation (%)	71.12	60.64	60.28	58.46	78.64	72.41	76.57
No SP annotation (%)	28.88	39.36	39.72	41.54	21.36	27.59	23.43
TREMBL annotation	30,116	18,106	25,478	19,447	24,376	19,992	208,698
TREMBL annotation (%)	88.90	84.72	80.74	83.83	93.44	90.91	84.21^a^
No TREMBL annotation (%)	11.10	15.28	19.26	16.17	6.56	9.09	15.78^a^
Pfam	24,000	12,686	19,056	13,283	20,514	15,665	16,938
Pfam annotated (%)	70.84	59.36	60.39	57.26	78.64	71.24	68.35
No Pfam annotated (%)	29.16	40.64	39.61	42.74	21.36	28.76	31.65
Total Pfam found	52,242	27,154	42,834	27,355	45,944	28,984	30,605
Pfam per gene	1.54	1.27	1.36	1.18	1.76	1.32	1.24

Abbreviations: ADIG, Acropora digitifera; AFEN, Amplexidiscus fenestrafer; ATEN, Actinia tenebrosa; DSPP, Discosoma sp., EPAL, Exaiptasia pallida; HVUG, Hydra vulgaris; NVEC, Nematostella vectensis.

a As the predicted proteome of *N. vectensis* is incorporated into the TREMBL protein database, a subset of TREMBL's database with *N. vectensis* predicted proteins removed was used instead.

Our assembly also resolved the complete mitochondrial genome for *A. tenebrosa* (GenBank accession MK291977), shown to be 20,691 bp long (Figure S1). The mitochondrion of *A. tenebrosa* was aligned to the recently completed *A. equina* mitochondrion (Wilding & Weedall, 2019), revealing identical gene order and protein‐coding sequence similarity. Nucleotide differences in the mitochondrion of *A. tenebrosa* and *A. equina* included a thymine insertion in the intergenic region between genes *ND6* and *CYTB* in *A. tenebrosa*, a transversion SNP was identified in the large RNA subunit, and a transition SNP was identified in the intergenic region between *COIII* and *COI* genes.

### 3.3 Gene family evolution

**Figure 1:**
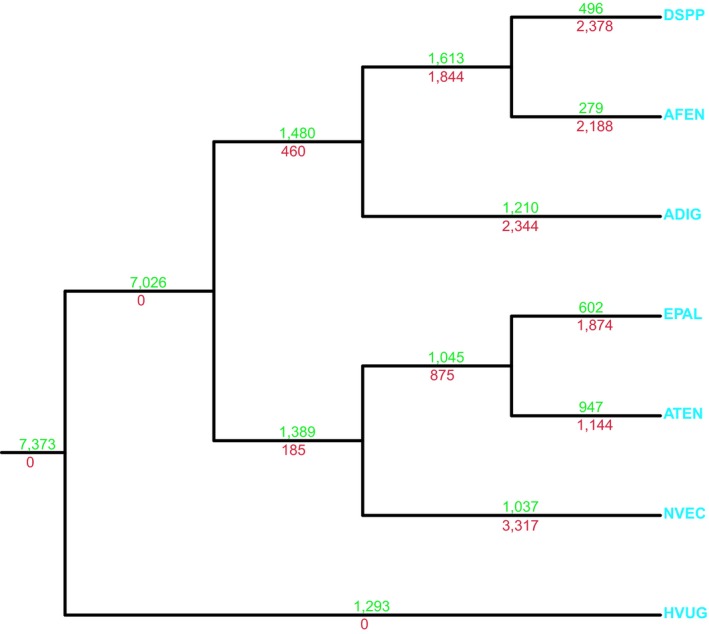
Comparative analysis of gene families within Cnidaria. Maximum‐likelihood protein tree generated to determine cnidarian phylogeny, all bootstrap support 100%. TTL gene family gains (green) and losses (red) are represented above and below branches, respectively. ADIG, *Acropora digitifera*; AFEN, *Amplexidiscus fenestrafer*; ATEN, *Actinia tenebrosa*; DSPP, *Discosoma* sp.; EPAL, *Exaiptasia pallida*; HVUG, *Hydra vulgaris*; NVEC, *Nematostella vectensis*

Manual curation and a phylogenomic characterization of seven Cnidarian species drove our investigation of Cnidarian gene turnover. Using 1,314 genes, we built a representative cnidarian species tree from all seven genomes (Figure 1). This species tree confirmed the phylogenetic position of *A. tenebrosa* with previously published species trees (Daly et al., 2017; Rodríguez et al., 2014; Wang et al., 2017). We found 7,373 gene families were shared among all cnidarian taxa investigated. An additional 7,026 gene families were gained in Anthozoa following their divergence from Medusozoa (*H. vulgaris*). In the actiniarian lineage (which includes *A. tenebrosa*, *E. pallida*, and *N. vectensis*), 1,389 and 185 gene families were gained and lost, respectively. Examination of the genome of *A. tenebrosa* found that 947 gene families (3,963 genes) were gained in this species following divergence from other sea anemone taxa investigated. In all cnidarians, lineage‐specific gene families have undergone a greater expansion compared with gene families shared among cnidarians (Table 3). This is most apparent in *A. tenebrosa* and *H. vulgaris*, with lineage‐specific gene families having a mean copy number of 4.18 and 4.99 genes, respectively. Additional novelty is observed with 6,705 (21.26%) singletons (lineage‐specific genes not in gene families) found in the gene models of *A. tenebrosa*. These results suggest significant gene family conservation across cnidarians, particularly in Anthozoa, but with lineage‐specific genes contributing to a significant proportion of the genome.

**Table 3:** Expansion of shared and lineage‐specific gene families in cnidarians

	ADIG	AFEN	ATEN	DSPP	EPAL	HVUG	NVEC
Total genes	33,878	21,372	31,556	23,199	26,087	21,990	24,780
Singletons	4,053	5,261	6,705	5,752	2,590	2,800	5,492
Singletons (%)	11.96	24.62	21.25	24.79	9.93	12.73	22.16
Total gene families	14,285	13,279	15,576	13,306	14,501	8,666	13,323
Total genes in gene families	29,825	16,111	24,851	17,447	23,497	19,190	19,288
Expansion	2.09	1.21	1.6	1.31	1.62	2.21	1.45
Lineage‐specific gene families	1,210	279	947	496	602	1,293	1,037
Lineage‐specific gene families (%)	8.47	2.1	6.08	3.73	4.15	14.92	7.78
Lineage‐specific genes	4,238	659	3,963	1,232	1,830	6,451	3,447
Expansion	3.5	2.36	4.18	2.48	3.04	4.99	3.32
Shared gene families	13,075	13,000	14,629	12,810	13,899	7,373	12,286
Shared gene families (%)	91.53	97.90	93.92	96.27	95.85	85.08	92.22
Shared genes	25,587	15,452	20,888	16,215	21,667	12,739	15,841
Expansion	1.96	1.19	1.43	1.27	1.56	1.73	1.29

Abbreviations: ADIG, *Acropora digitifera*; AFEN, *Amplexidiscus fenestrafer*; ATEN, *Actinia tenebrosa*; DSPP, *Discosoma* sp.; EPAL, *Exaiptasia pallida*; HVUG, *Hydra vulgaris*; NVEC, *Nematostella vectensis*.

**Figure 2:**
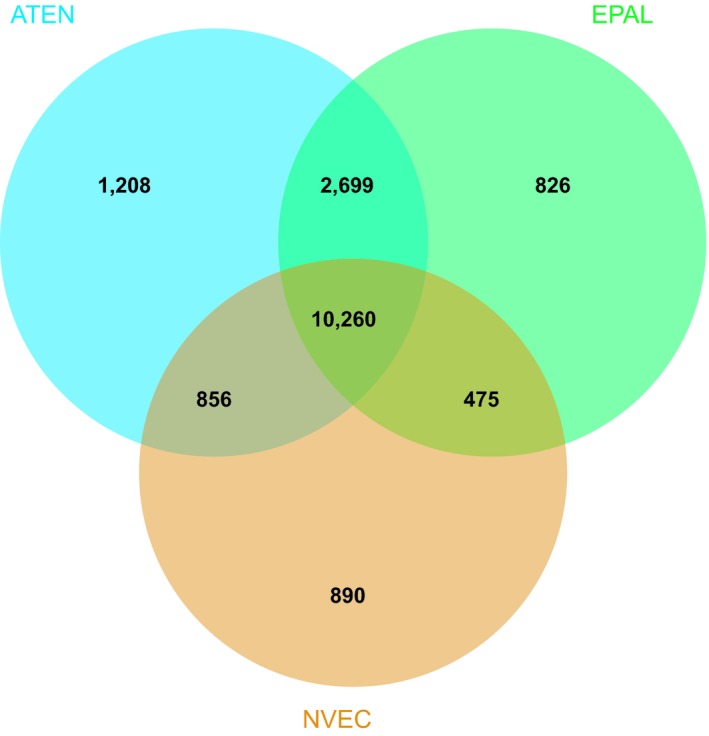
Comparative analysis of gene families among Actiniarians. Venn diagram highlighting orthologous genes between Actiniarian genomes. EPAL, *Exaiptasia pallida*; NVEC, *Nematostella vectensis*; and ATEN, *Actinia tenebrosa*

A closer examination of gene families within Actiniaria revealed 10,260 orthologs shared across the three actiniarian genomes investigated (Figure 2). These 10,260 actiniarian orthologs, however, do not exhibit any GO term enrichment. Five GO terms, including nematocyst (GO: 0042151; Table S3), were over‐represented in the predicted protein sequences from the 1,208 genes unique to *A. tenebrosa*. This highlights that a significant proportion of genes unique to *A. tenebrosa* have roles related to envenomation. Although all actiniarians are venomous, we observe, therefore, the first expansion of lineage‐specific genes is related to venom delivery.

**Figure 3:**
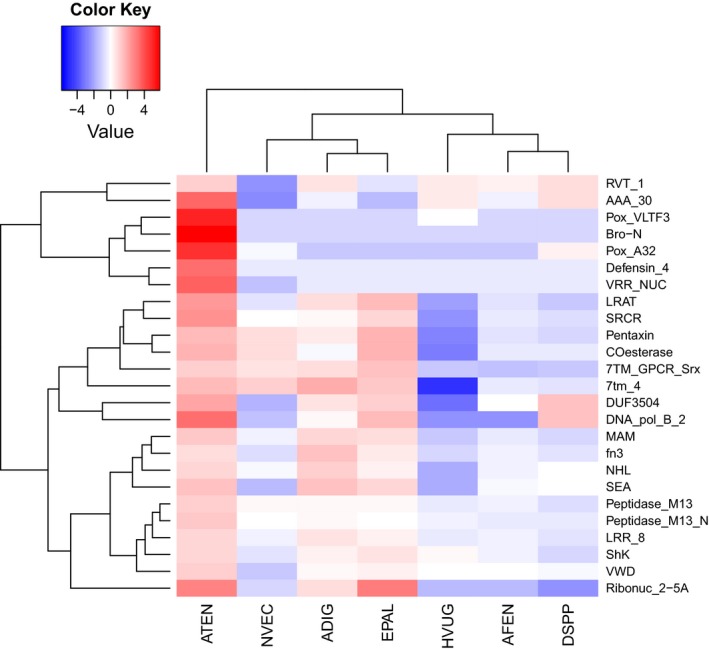
Protein domain enrichment across Cnidaria. Heat map of Pfam domains enriched in *Actinia tenebrosa*. Abundance of Pfam domains in cnidarians log2 and median centered. ADIG, *Acropora digitifera*; AFEN, *Amplexidiscus fenestrafer*; ATEN, *Actinia tenebrosa*; DSPP, *Discosoma* sp.; EPAL, *Exaiptasia pallida*; HVUG, *Hydra vulgaris*; NVEC, *Nematostella vectensis*

To better understand the evolution of protein domains across cnidarian genomes, we also investigated Pfam domain enrichment. Using a Fisher exact test, 25 Pfam domains were significantly enriched in *A. tenebrosa*, in comparison with other cnidarian genomes (Figure 3). Enrichment of ShK and Defensin_4 domains underpinned much of the expansion of toxin‐related genes in *A. tenebrosa*. Both ShK and Defensin_4 domains are associated with potassium channel‐blocking toxins in sea anemones, specifically sea anemone type 1 potassium channel toxin (KTx) and type 3 (BDS‐LIKE) KTx, respectively (Castañeda et al., 1995).

**Figure 4:**
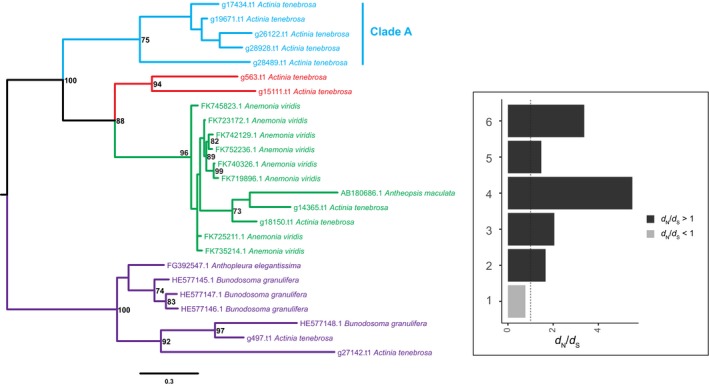
Maximum‐likelihood tree with midpoint root depicting relationships among sea anemone type 3 (BDS‐LIKE) KTx coding sequences. Bootstrap values after 1,000 iterations are shown next to nodes, values under 70% not reported. The GenBank accession numbers for the protein‐coding gene used in this phylogenetic analysis are described in Sunagar and Moran (2015). A corresponding bar plot is provided which shows the computed dN/dS value for orthologs and paralogs. 1 = Actiniaria orthologs, 2 = *Anemonia viridis* paralogs, 3 = *Actinia tenebrosa* paralogs, 4 = *Bunodosoma granulifera* paralogs, 5 = *Actinia tenebrosa* Clade A paralogs (Clade A), and 6 = *Actinia tenebrosa* paralogs diverged

With evidence supporting that genetic innovations in the genome of *A. tenebrosa* are related to venom, we further investigated its total and toxin‐like gene (TTL) complement. Overall, we identified 113 TTL gene families in *A. tenebrosa* (Table S4). Manual curation of TTL genes revealed that sea anemone type 3 (BDS‐LIKE) KTx family is the most highly expanded TTL gene family (15 copies, 11 of which are full‐length sequences). A phylogeny of sea anemone type 3 (BDS‐LIKE) KTx was generated from these full‐length sequences (Figure 4), as well as functionally characterized sequences from other sea anemones (Jouiaei, Sunagar, et al., 2015; Sunagar & Moran, 2015). The 11 *A. tenebrosa* sequences clustered into four distinct clades, one of which includes only *A. tenebrosa* paralogs (Clade A). This suggests a process of concerted‐like evolution. Investigation into the genomic localization of the 11 *A. tenebrosa* sequences revealed no presence of tandem duplication, a common mechanism observed during concerted evolution. Furthermore, the sequence identity among *A. tenebrosa* sea anemone type 3 (BDS‐LIKE) KTx paralogs is highly divergent at 34.2% and 43.5% at the nucleotide and protein level, respectively.

**Figure 5:**
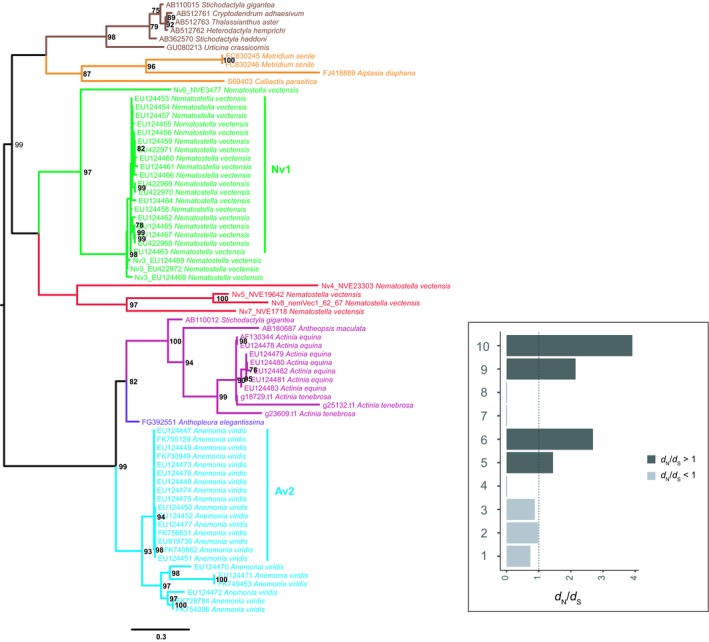
Maximum‐likelihood tree with midpoint root depicting relationships among NaTx coding sequences. Bootstrap values after 1,000 iterations are shown next to nodes, values under 70% not reported. The GenBank accession numbers for the protein‐coding gene used in this phylogenetic analysis are described in Sunagar and Moran (2015) and Sachkova et al. (2019). A corresponding bar plot is provided which shows the computed dN/dS value for orthologs and paralogs. 1 = Actiniaria orthologs, 2 = *Actinia* orthologs, 3 = *Nematostella vectensis* paralogs, 4 = *Actinia equina* paralogs, 5 = *Actinia tenebrosa* paralogs, 6 = *Anemonia viridis* paralogs, 7 = Nv1 paralogs, 8 = Av2 paralogs, 9 = *Nematostella vectensis* paralogs diverged, and 10 = *Anemonia viridis* paralogs diverged

The sea anemone sodium channel inhibitory toxin family (NaTx) has also previously been shown to evolve via concerted evolution in multiple different sea anemone species (Moran et al., 2008). Here we generated a phylogeny for the NaTx gene family (Figure 5), using sequences from a previously published alignment (Jouiaei, Sunagar, et al., 2015; Sunagar & Moran, 2015), as well as newly identified sequences from *N. vectensis* (Nv4, Nv5, Nv6, Nv7, and Nv8; Sachkova et al., 2019). The phylogeny of NaTx gene family confirmed evidence of concerted evolution in multiple species, with paralogs clustering strongly together in *N. vectensis*, *A. viridis*, and *A. equina*. Three copies of NaTx were identified in *A. tenebrosa*, with two copies clustering together and another paralog clustering with *A. equina* sequences. The three *A. tenebrosa* paralogs share 61.1% and 52.9% sequence similarity at the nucleotide and protein level, respectively. Additionally, the genome of *A. tenebrosa* revealed no evidence of tandem duplication for the three NaTx paralogs.

**Figure 6:**
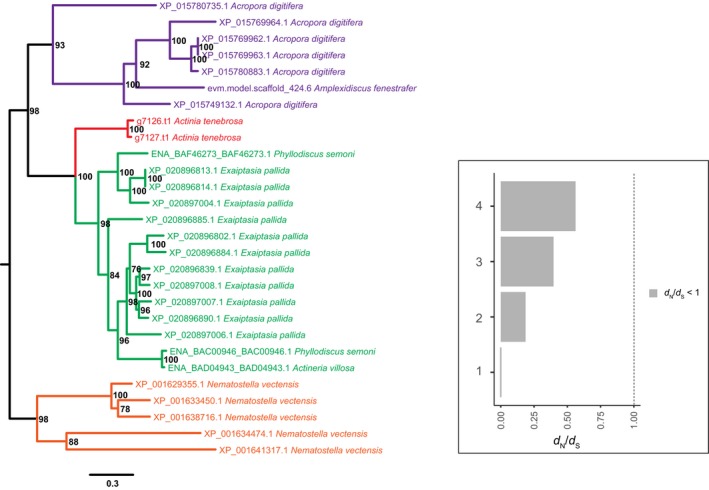
Maximum‐likelihood tree with midpoint root depicting relationships among MACPF coding sequences. Bootstrap values after 1,000 iterations are shown next to nodes, values under 70% not reported. The European Nucleotide accession numbers for the protein‐coding genes are reported. A corresponding bar plot is provided which shows the computed dN/dS value for orthologs and paralogs. 1 = Anthozoan orthologs, 2 = *Nematostella vectensis* paralogs, 3 = *Exaiptasia pallida* paralogs, and 4 = *Acropora digitifera* paralogs

**Figure 7:**
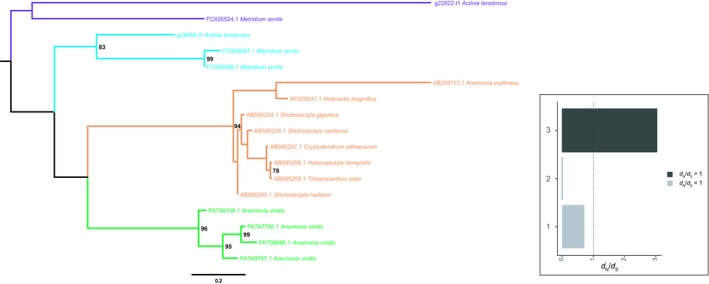
Maximum‐likelihood tree with midpoint root depicting relationships among sea anemone type 1 KTx coding sequences. Bootstrap values after 1,000 iterations are shown next to nodes, values under 70% not reported. The GenBank accession numbers for the protein‐coding gene used in this phylogenetic analysis are described in Sunagar and Moran (2015). A corresponding bar plot is provided which shows the computed dN/dS value for orthologs and paralogs. 1 = Actiniaria orthologs, 2 = *Metridium senile* paralogs, and 3 = *Anemonia viridis* paralogs

Investigating the phylogenetic histories of cnidarian MACPF gene family also revealed evidence of concerted‐like evolution (Figure 6). This included two paralogs of *A. tenebrosa* MACPF sequences clustering together. Genomic localization further revealed these sequences evolved through tandem duplication (Figure S2). Evidence of concerted evolution was also revealed with *A. tenebrosa* MACPF sequences being highly homogenous, sharing 94.3% and 92.8% similarly at the nucleotide and protein level, respectively. In fact, clustering of paralogs of MACPF was observed in the majority of the anthozoan genomes investigated, including all sea anemones. Multiple tandem duplications were observed in *E. pallida*; however, this was not consistent in all sea anemones with *N. vectensis* paralogs not adjacent to each other in the genome. We also found evidence of concerted‐like evolution in the sea anemone type 1 KTx family (Figure 7). While we did not observe this for *A. tenebrosa* paralogs, this process was observed for *A. viridis* paralogs.

While concerted‐like evolution appears to be a consistent pattern of TTL genes families in sea anemones, similar pattern is also observed broadly in cnidarians florescent protein (FP) family (Figure S3), a nontoxin gene family. Combining published data (Alieva et al., 2008; Ikmi & Gibson, 2010), with the genomic datasets from this study, we observed a consistent pattern of paralogs clustering together. This is observed for *A. tenebrosa* FP paralogs that cluster together in a clade consisting of other sea anemone chromoprotein sequences. While these sequences from *A. tenebrosa* appear to be evolving via concerted evolution, this appears to not be reliant on tandem duplication. While the *A. tenebrosa* paralogs sequence have escaped tandem duplication, they maintain a high level of sequence identity of 95.6% and 93.6% at the nucleotide and protein level, respectively. A similar pattern is also observed in other Hexacorallia taxa including *N. vectensis* and *A. fenestrafer*.

### 3.4 Selection patterns on toxin gene families

In this study, we further explored the evolutionary histories of TTL gene families to provide insights into the selective pressures acting on them (Table S5). Here we report evidence of purifying acting on all TTL gene families. Given the evidence of concerted‐like evolution acting on many of the gene families investigated, we tested the selective pressures of paralogs where possible. In *A. tenebrosa*, paralogs from the sea anemone type 3 (BDS‐LIKE) KTx (Figure 4; d*N*/d*S* = 2.0515) gene family revealed evidence of diversifying selection. Similarly, in *A. viridis*, sea anemone type 3 (BDS‐LIKE) KTx paralogs also appear to be evolving under diversifying selection (Figure 4; d*N*/d*S* = 1.6665). We further explored the selective pressures acting on NaTx paralogs. Paralogs from *A. tenebrosa* and *A. viridis* appear to be evolving under diversifying selection, with a d*N*/d*S* ratio of 1.4438 and 2.6865, respectively. In *A. equina*, however, we cannot confirm diversifying selection acting on paralogs. Differences in selective pressures among a subset of NaTx orthologs were also observed, with orthologs from *Actinia* genus (d*N*/d*S* = 0.9825) and appear to be evolving under a relaxed rate of purifying selection compared to among actiniarian orthologs (d*N*/d*S* = 0.7397). *Nematostella vectensis* NaTx paralogs had a d*N*/d*S* ratio (0.881) consistent with the action of purifying selection. Additionally, all sea anemone type 1 KTx paralogs are inferred to be evolving under purifying selection, with the exception of *A. viridis* (Figure 7; d*N*/d*S* = 3.0389).

Divergent evolutionary histories were also observed among paralogs of gene families that appear to be evolving through a process of concerted‐like evolution. Specifically, in NaTx and sea anemone type 3 (BDS‐LIKE) KTx gene families, some paralogs are evolving through a process of concerted evolution and others are escaping this process. This is observed for *N. vectensis* and *A. viridis* NaTx paralogs, and *A. tenebrosa* sea anemone type 3 (BDS‐LIKE) KTx paralogs. For *N. vectensis*, no evidence of positive selection could be inferred among the highly homogenous Nv1 sequences (Figure 5); however, paralogs that have escaped this homogenization are inferred to be evolving under diversifying selection (Nv3‐8; d*N*/d*S* = 2.1451). This is also observed for *A. viridis* NaTx paralogs. While we could not infer the selective pressures acting on the highly homogenous Av2 sequences, those that have diverged are undergoing diversifying selection (d*N*/d*S* = 3.9116). In *A. tenebrosa*, some sea anemone type 3 (BDS‐LIKE) KTx sequences paralogs also show strong clustering (Figure 4 Clade A), while other paralogs cluster with sequences from other sea anemones. While both sets of paralogs are evolving under diversifying selection (Clade A d*N*/d*S* = 1.4761, diverging paralogs in *A. tenebrosa* d*N*/d*S* = 3.3732), those that have diverged show pronounced signatures of diversifying selection. Additionally, we also have evidence of concerted‐like evolution for the FP and MACPF gene families; however, we did not observe any paralogs escaping this process (Figures 6 and S3).

## 4 DISCUSSION

Here we present a draft genome assembly and annotation of *A. tenebrosa*. This complete draft assembly is the first from any species of the superfamily Actinioidea. Overall, the assembly was of similar quality and completeness to currently published anthozoan genomes (Baumgarten et al., 2015; Chapman et al., 2010; Putnam et al., 2007; Shinzato et al., 2011; Wang et al., 2017), verifying its suitability for comparative genomic studies. Insights into the evolution of gene families across Cnidaria revealed significant conservation among anthozoan species, with the many gene families gained in either the last common ancestor of Cnidaria or Anthozoa. Notably, all anthozoans used in this study are from Hexacorallia, highlighting a high conservation of gene families shared among this subclass. This is consistent with previous studies that have suggested that this shared gene set plays an important role in the evolution of traits essential to Hexacorallia taxa, including symbiosis with dinoflagellates, stress response, and delivery of venom (Baumgarten et al., 2015; Rachamim et al., 2015; Wang et al., 2017).

The *A. tenebrosa* genome is the most gene dense among cnidarians, with only *E. pallida* having a smaller genome and only *A. digitifera* having more protein‐coding genes. However, flow cytometry revealed that the genome size of *A. equina* is larger (~520 Mb) than that predicted here ~255 Mb (Adachi, Miyake, Kuramochi, Mizusawa, & Okumura, 2017). One hypothesis for the discrepancy observed between estimated genome sizes may be associated with repeat regions that have not been fully captured in our assembly. The *A. tenebrosa* genome also contained a higher proportion of lineage‐specific genes compared with other cnidarian genomes. Previous studies have identified this pattern in species from the superfamily Actinioidea, particularly those genes that encode for peptide toxins (Prentis et al., 2018; Surm et al., 2019). It is shown that there is relatively little overlap of toxin genes among cnidarian species and that a high proportion are restricted to specific lineages (Rachamim et al., 2015; Surm et al., 2019). In addition, many lineage‐specific toxins from *A. tenebrosa* have expression restricted to acrorhagi, a novel structure used for envenomation. These data support the hypothesis that novel genes are expressed in novel morphological structures. Evidence in support of this hypothesis in other cnidarian species is equivocal. For example, although *Nematostella*‐specific genes comprise a significant proportion of genes expressed in the nematostome, a novel structure only found in this genus, many of these genes were also expressed in tissues common to all sea anemone species (Babonis, Martindale, & Ryan, 2016).

The origin of new genes is considered to be an important source of evolutionary novelty, by providing the substrate upon which natural selection can act. New genes may be formed through multiple processes, ranging from gene duplication through exon shuffling to de novo gene formation (Kaessmann, 2010; McLysaght & Hurst, 2016; Tautz & Domazet‐Lošo, 2011). Genes created through these processes produce copies of a gene that are identical to the ancestral sequence or generate genes with novel sequences that are restricted to specific lineages (Capra, Pollard, & Singh, 2010). Here, we have revealed that lineage‐specific gene families undergo increased rates of gene duplication compared with gene families shared among cnidarian orders. This suggests that following the formation of new genes in cnidarian taxa, repeated duplication events occur. However, this also suggests that few new genes arise through de novo gene evolution in cnidarians, as genes generated through this mechanism have been reported to undergo limited gene duplications (Schlötterer, 2015).

Sea anemones, and in particular species from the superfamily Actinioidea, are an important group used to understand the evolution of toxins. Our results support this, with gene families encoding peptide toxins enriched in *A. tenebrosa* relative to sea anemones from other superfamilies. For example, genes involved in venom production (peptide toxins) or delivery (cnidocyte) are associated with the nematocyst GO term, which are significantly over‐represented in the gene families restricted to *A. tenebrosa*. This GO term was not enriched for toxin genes restricted to *N. vectensis* or *E. pallida*. This result, however, may be a consequence of ascertainment bias as the majority of toxins characterized in actiniarians to date have been identified in the superfamily, Actinioidea (206 of the 236 cnidarian toxins; Prentis et al., 2018). Furthermore, difference in genome assemblies and annotations methods may also contribute to differences observed in gene family evolution among cnidarian genomes.

We propose that the major contributor to the evolution of new genes in cnidarians is through a process of gene duplication. Significant expansions of neurotoxins are observed in *A. tenebrosa*. This was evident from the increased copy number of Pfam domains (ShK and Defensin_4) which are associated with neurotoxins that modulate potassium ion channels. The Defensin_4 domain is associated with the sea anemone type 3 (BDS‐LIKE) potassium channel toxin family, and both the gene family and protein domain are restricted to Actinioidea (Diochot, Schweitz, Béress, & Lazdunski, 1998). This is supported by evidence of the sea anemone type 3 (BDS‐LIKE) potassium channel toxin family identified to be gained in the 947 Actinioidean‐specific gene families (Figure 1). Furthermore, sea anemone type 3 (BDS‐LIKE) KTx appears to be the most highly duplicated toxin‐encoding gene in *A. tenebrosa*.

In this study, we explored the selective pressures acting on orthologs and paralogs in TTL gene families to investigate the adaptative evolution of lineage‐specific duplications. We revealed repeated evidence of paralogs evolving at an accelerated rate compared with orthologs. Our findings identified that TTL paralogs often cluster together, suggesting recent duplications undergo accelerated rates of nonsynonymous substitutions, whereas nucleotide variation in ancient duplications is driven by selective forces that limit deleterious mutations (purifying selection). This pattern is supported by the work of Sunagar and Moran (2015) who observed this pattern of divergent selective pressures among ancient and young venomous lineages. The authors suggest that the evidence of diversifying selection acting on younger venomous lineages is driven by recent duplications allowing for the adaptations to an ecological niche. In ancient venomous lineages, however, TTL genes encoding toxins that resulted from ancient duplications events are dominated by purifying selection to limit deleterious mutations. This suggests that toxins in ancient venomous lineages have become specialized to their ecological requirements. Our study supports these findings, and we further suggest that lineage‐specific duplications may drive the adaptive evolution of toxins in ancient venomous lineages required to meet their ecological and life history requirements. This pattern was not conserved for all TTL gene families, however, with MACPF paralogs and *N. vectensis* NaTx paralogs evolving under purifying selection. This may be due to members of both gene families instead evolving via a process consistent with the action of concerted evolution.

Diverse evolutionary trajectories exist following gene duplication including pseudogenization, neofunctionalization, and subfunctionalization. An additional trajectory includes conservation which can be driven through a process of concerted evolution. Concerted evolution is the homogenization of paralogs that results in sequence similarity greater within species compared to between species (Liao, 1999; Nei & Rooney, 2005). This homogenization is typically attributed to gene conversion or unequal‐crossing over (Brown, Wensink, & Jordan, 1972; Eickbush & Eickbush, 2007; Szostak & Wu, 1980). Here we observe concerted‐like evolution in multiple TTL gene families including sea anemone types 1 and 3 (BDS‐LIKE) KTx, NaTx, MACPF, and the nontoxin gene family FP. Whether the concerted‐like evolution observed is through lineage‐specific duplications or concerted evolution remains elusive.

Concerted evolution of a sea anemone toxin gene family has previously been reported in multiple species (Moran, Genikhovich, et al., 2012; Moran et al., 2008). Nv1, a member of NaTx TTL gene family, is the major adult venom component in *N. vectensis* Nv1 has evolved via concerted evolution. This is supported by the evidence of Nv1 copies being encoded by a cluster of at least 12 highly conserved sequences (Moran, Genikhovich, et al., 2012; Moran et al., 2008). This is further supported in the NaTx phylogeny we generated in this study, with Nv1 sequences clustering strongly together (Figure 5). From our selection analyses, we could not infer that these highly homogenous Nv1 sequences are evolving under diversifying selection. Divergently, the *N. vectensis* paralogs that escaped this homogenization are inferred to be evolving under diversifying selection, which consists of Nv3‐8. Recent experimental evidence supports the adaptive evolution of these paralog escaping the process of concerted evolution (Sachkova et al., 2019). This is evident with Nv4 and Nv5 paralogs expression being mostly restricted to early life stages compared with Nv1, suggesting neofunctionalization or subfunctionalization. The Nv4 and Nv5 paralogs also exhibit divergent activity being highly toxic to fish, compared with Nv1 which has greater activity against arthropods (Sachkova et al., 2019). Indeed, a similar pattern is also observed in *A. viridis* NaTx paralogs with the Av2 copies being highly similar and other copies escaping this homogenization. These escaped paralogs are also inferred to be evolving under diversifying selection. While evidence supports that both Nv1 and Av2 are evolving through a process of gene conversion or unequal‐crossing over consistent with concerted evolution (Moran et al., 2008), the escaped paralogs, however, may have resulted from lineage‐specific duplications (Sachkova et al., 2019).

Overall, our phylogenetic analyses provide repeated evidence of paralogs clustering closer together than orthologs for multiple TTL gene families in cnidarians. Whether this occurs through a process of concerted evolution (gene conversion or unequal‐crossing over) or lineage‐specific duplications is unclear, especially given that a combination of both processes may be occurring in parallel. We propose that concerted evolution is an important process in the evolution of ancient actiniarian venom, occurring in gene families recruited into the venom of at least last common ancestor. Subsequently, lineage‐specific duplications allow paralogs to escape the homogenizing process associated with concerted evolution, with selection driving these new duplicates to undergo neofunctionalization or subfunctionalization.

In venomous animals, biochemical and morphological innovations result in phenotypic adaptations, such as toxin peptides and an envenomation system. Although the cnidarian envenomation system is largely conserved across this phylum, our analysis revealed duplication events in gene families enriched in *A. tenebrosa* include many nematocyte‐related proteins such as toxin peptides. We propose that the genome sequence of *A. tenebrosa* will aid future research to improve our understanding of Actinioidean innovations involved in venom production and its delivery.

## CONFLICT OF INTEREST

We declare there are no conflicts of interest.

## AUTHOR CONTRIBUTIONS

All authors conceived and designed the project. JMS, PJP, and AnP collected organism samples. JMS performed DNA extraction. AlP assembled genome, and ZKS annotated genome. JMS performed comparative genomics and phylogenetic analysis. JMS led the draft of the manuscript with contributions from all authors. All authors read and approved the final version.

## Data Availability

A description and overview of the project are available under the BioProject accession number PRJNA505921. A description of the complete mitochondrion is available through GenBank accession number MK291977.
