# Supplementary figures and images for: The draft genome of Actinia tenebrosa reveals insights into toxin evolution
Source: Ecol Evol. 2019 Sep 18;9(19):11314–28. doi: 10.1002/ece3.5633 (PMC6802032; doi:10.1002/ece3.5633)

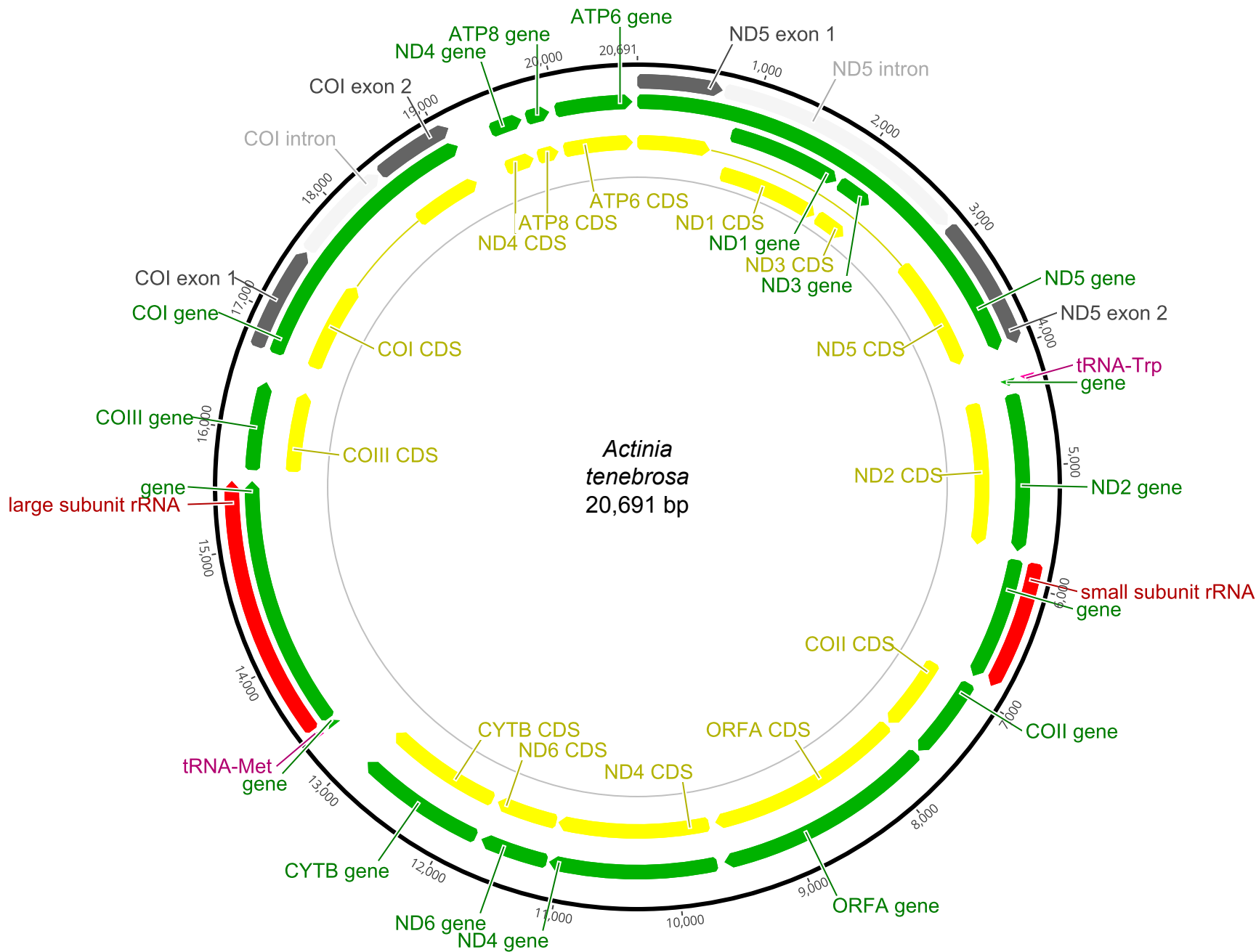

Supplement: Supplementary file 1 [file ECE3-9-11314-s001.pdf]

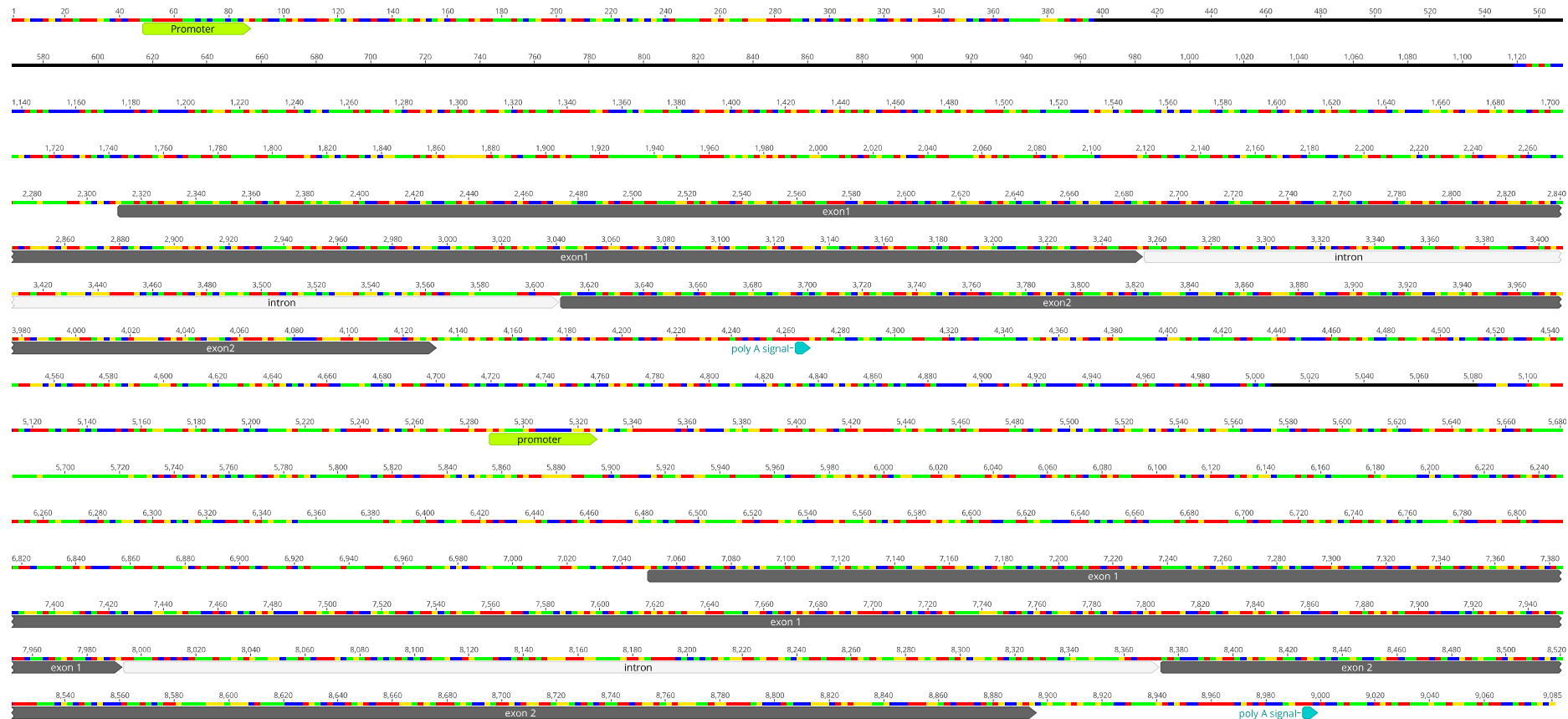

Supplement: Supplementary file 2 [file ECE3-9-11314-s002.pdf]

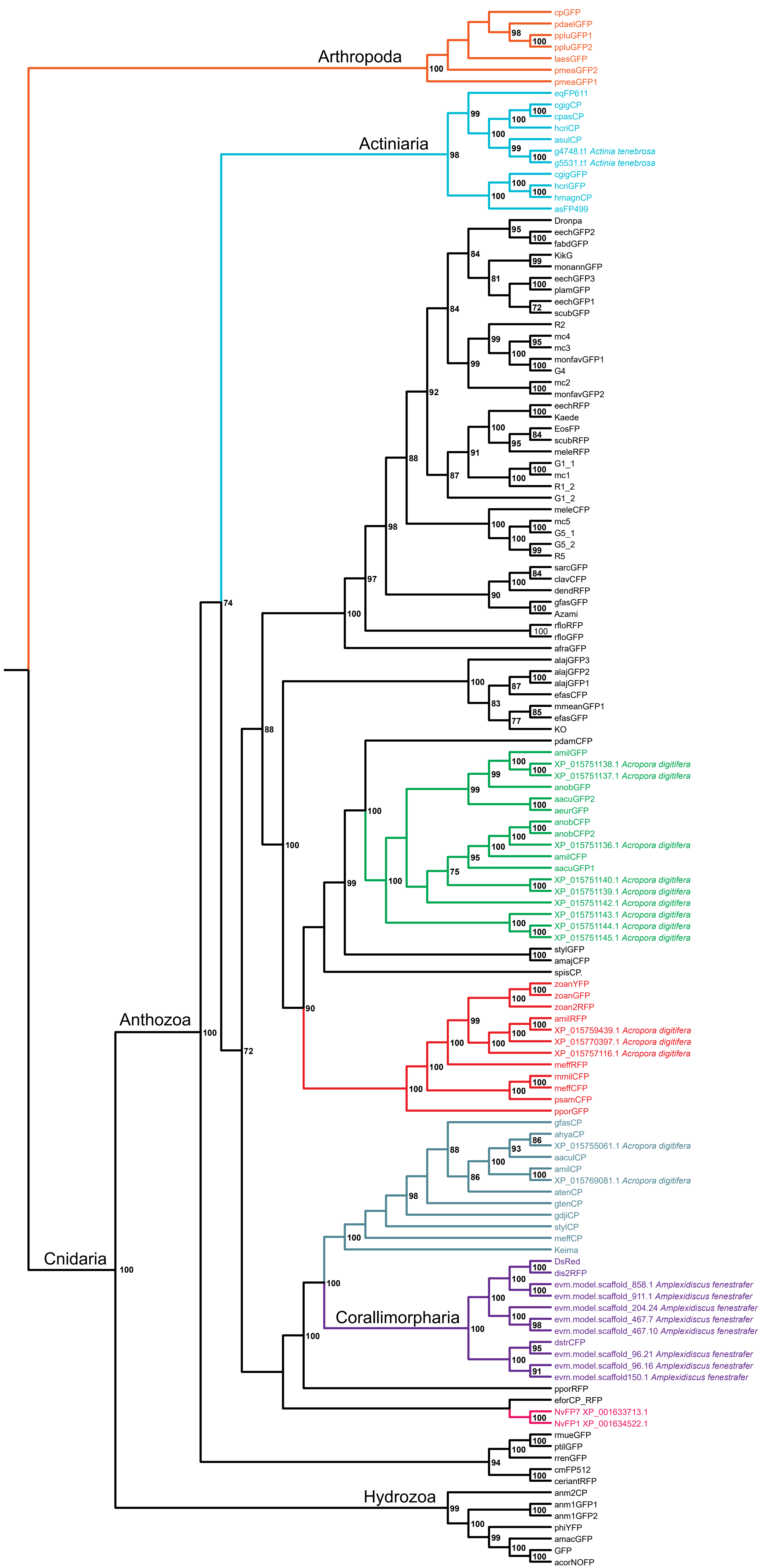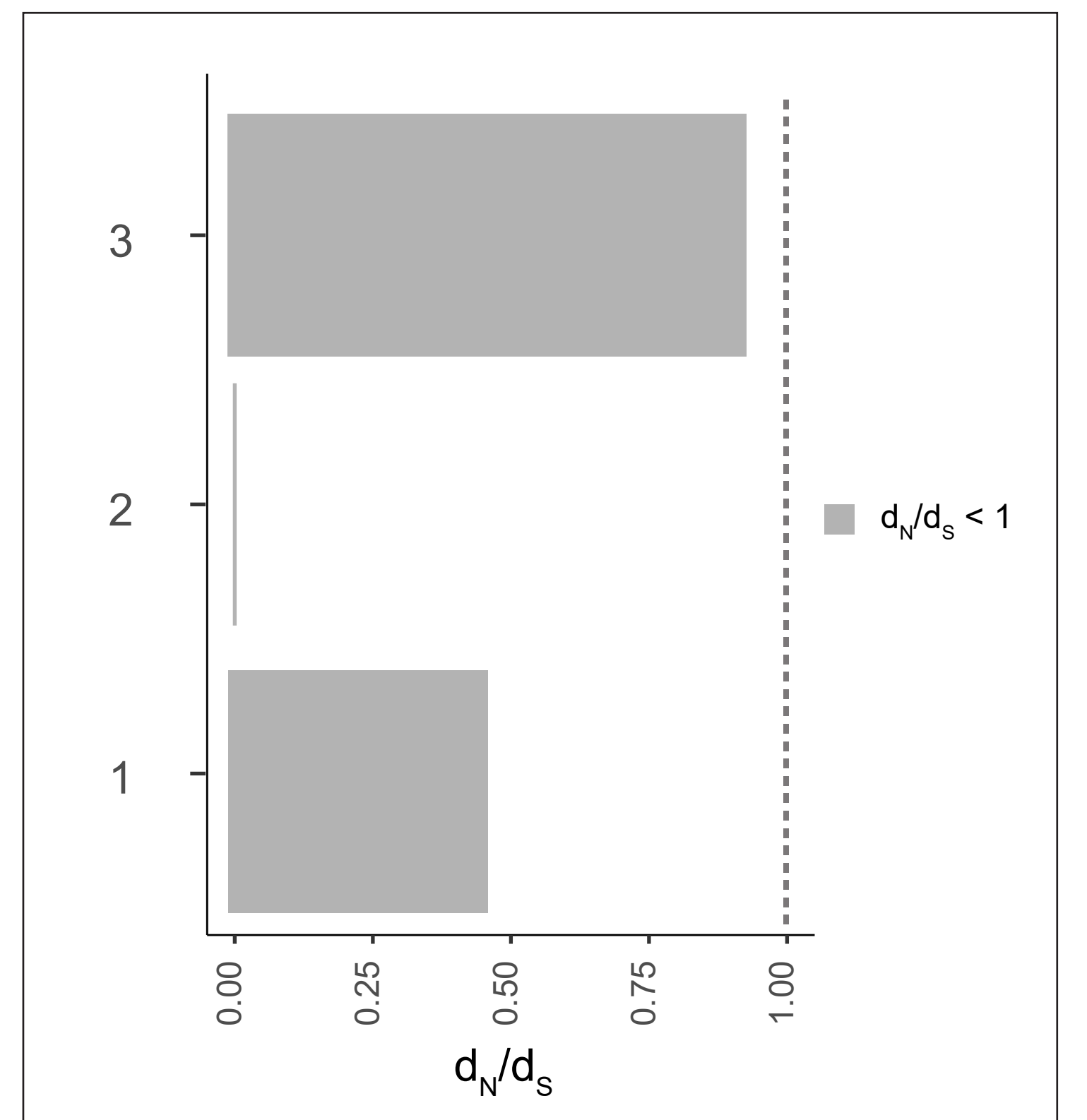

Supplement: Supplementary file 3 [file ECE3-9-11314-s003.pdf]
