# Supplementary material for: The draft genome of Actinia tenebrosa reveals insights into toxin evolution
Source: Ecol Evol. 2019 Sep 18;9(19):11314–28. doi: 10.1002/ece3.5633 (PMC6802032; doi:10.1002/ece3.5633)
Supplement: Supplementary file 4 [file ECE3-9-11314-s004.docx]

**Supplementary Table 1. Illumina raw reads metrics used to generate the draft genome of *Actinia tenebrosa***

| Insert | Read length(bp) | Clean reads | Clean bases (bp) | Q20(%) | GC(%) |
| --- | --- | --- | --- | --- | --- |
| 170bp | 100 | 323,912,222 | 32,391,222,200 | 97.39 | 41.57 |
| 2kbp | 100 | 313,781,050 | 31,378,105,000 | 97.20 | 40.27 |
| 500bp | 100 | 324,434,076 | 32,443,407,600 | 96.15 | 39.22 |
| 5kbp | 100 | 323,546,606 | 32,354,660,600 | 97.56 | 39.91 |

**Supplementary Table 2. Repeats breakdown masked in the Actinia tenebrosa genome**

| **Class** | **Nt masked** | **% masked** |
| --- | --- | --- |
| **SINE** | 2,657,682 | 1.29 |
| **RC** | 407,575 | 0.20 |
| **SINE-like** | 26,191 | 0.01 |
| **Unknown** | 8,816,058 | 4.29 |
| **MITE** | 21,217,408 | 10.31 |
| **LINE** | 1,049,346 | 0.51 |
| **LTR** | 2,311,971 | 1.12 |
| **DNA** | 3,643,579 | 1.77 |

**Supplementary Table 3. Gene set enrichment analysis of gene ontologies from genes unique to *Actinia tenebrosa* within actiniarian**

| Gene ontology ID | Name | Namespace | *P-*value |
| --- | --- | --- | --- |
| GO:0003964 | RNA-directed DNA polymerase activity | Molecular function | 8.5*e*^-06^ |
| GO:0005044 | scavenger receptor activity | Molecular function | 8.4 *e*^-04^ |
| GO:0015074 | DNA integration | Biological process | 0.0027 |
| GO:0060107 | annuli extracellular matrix | Cellular component | 0.032 |
| GO:0042151 | nematocyst | Cellular component | 0.032 |

**Supplementary Table 4.** Cnidarian Toxin and toxin-like gene families and copy number

See attached

**Supplementary Table 5.** Detecting diversifying selection using site models implemented in CODEML for the toxin and non-toxin gene families from cnidarian genomes

See attached

**Supplementary Figure 1.** Gene order in *Actinia tenebrosa* mitochondrial DNA (20,691 bp). Figure produced in Geneious 9.1.6.

**Supplementary Figure 2.** Tandem duplication of *Actinia tenebrosa* MACPF. Figure produced in Geneious 9.1.6.

**Supplementary Figure 3.** Maximum‐likelihood tree with midpoint root depicting relationships among FP coding sequences. Bootstrap values after 1,000 iterations are shown next to nodes, values under 70% not reported. The GenBank accession numbers for the protein-coding gene used in this phylogenetic analysis are described in Alieva et al. (2008). A corresponding bar plot is provided which shows the computed dN/dS value for orthologs and paralogs. 1 = Cnidaria orthologs, 2 = *Acropora digitifera* paralogs*,* 3 = Amplexidiscus fenestrafer paralogs
